# The Aniline Dyes

**Published:** 1891-06-13

**Authors:** 


### THERAPEUTICS.

### THE ANILINE DYES.

As histological staining reagents, the aniline dyes have become familiar to almost every medical man; it is only very recently, however, that they have taken a place as therapeutical substances. They appear to have as wide a range of usefulness in medicine as is claimed for many recently-introduced remedies.

As outward applications, Professor Stilling has shown they are efficient antiseptics, fulfilling many of the conditions of the ideal antiseptic, which he defines as a substance capable of destroying bacteria while being innocuous to the human organism; very diffusible not only as regards its action on the tissues, but also so as to be able to spread through a whole colony of micro-organisms.

In Professor Stilling's experiments, the violet dyes, chiefly methyl-violet, were employed. He found that a solution of two per thousand added to paste made with wheaten meal, prevented its turning sour, however long it was kept. To test its antiseptic properties he decided to use moulds and the bacteria of putrefaction as being more resistant than pathogenic micro-organisms, and of all moulds the mucor stolonifer was chosen on account of its very rapid growth and great resistance. The mucor stolonifer was sown upon small rolls of bread, some of which were soaked in a 1·500 and 1·1000 solution of methyl-violet, some in water only. On the latter a full and luxuriant growth appeared in twenty-four hours, whilst on the former there was no sign of growth, even after fourteen days. From these and other experiments, it appears that methyl-violet in suitable strengths acts as a complete check to the development of bacteria of putrefaction; and as they are more resistant than pathogenic organisms, it must be taken for granted that the latter would very easily succumb to their action. This was confirmed by direct experiment with staphylococcus pyogenes aureus. Various dyes, such as fuchsin, methyl-blue, rhodamin vesuvin, and many others were experimented with, but the most efficient and practically useful was found to be methyl-violet, to which Stilling has given the name "pyoctanin."

Further experiments were made on rabbits' eyes, as his original purpose was to find an antiseptic for ophthalmic use. A rabbit's eye was inoculated with staphylococcus pyogenes aureus, causing a severe hypopion. Some methyl-violet solution was then dropped on it, causing a deep staining of the whole extent of the external wound, and also of the flaky collections of pus in the anterior chamber, putting a stop to the suppurative process. Stilling has obtained in his clinic most successful results in the treatment of corneal ulcers which had hitherto resisted all the usual remedies, and is of opinion that the methyl-violet treatment for these cases will supersede that by the galvano-cautery. He has also used it very successfully in a number of other external eye diseases, such as blepharitis, conjunctivitis, phlyctœna, and eczema of the lids; and in internal eye diseases, such as keratitis parenchymatosa, iritis serosa, old-standing cases of choroiditis, and even in one case of sympathetic ophthalmia. He has also found it useful in varicose ulcers.

Stilling suggests that, since it has been shown by his experiments that one can inject relatively large quantities into the peritoneal and pleural cavities without injury, and as animals can eat also without injury large quantities of the dye, it is also conceivable that the methyl-violet treatment of purulent pleurisy and peritonitis, typhoid and dysenteric ulcers, &c., is not beyond the bounds of possibility.

Methyl-violet and safranin solutions have been employed by MM. Hugounencq and Eraud in various cases. They found in their preliminary experiments with cultures of staphylococci and gonococci that the virulence of these micro-organisms was attenuated when left in contact with dilute solutions of either of these substances, and that by fairly concentrated solutions the vitality of the microbes was impaired and possibly destroyed. They obtained very good results in gonorrhœa by the injection of 1 in 100 and 1 in 150 solutions from 10 to 15 times a-day. If only three or four injections are given in the day neither the character nor the duration of the discharge is influenced in any way.

Maragliano has used methyl-violet in laryngeal tuberculosis with doubtful effect. The microbal views of the pathology of cancer have naturally led to the employment of germicidal remedies, and Stilling's experiments would point to methyl-violet as an efficient and suitable agency. Professor Mosetig-Moorhof's "dyeing method" of treating malignant growths which are unsuitable for operation may, perhaps, depend on the antiseptic properties of methyl-violet, although he was led to adopt his method as far back as 1883 from the fact that the growth of neoplasms depends on the vitality of the cell nuclei, which greedily absorb pigments. He first employed anilinum trichloratum, but since last year has used methyl-violet. One of his cases, a woman aged 66, on coming into his hospital appeared to be dying of starvation. She had a tumour, larger than a man's fist, on her chin, and occupying more than the anterior half of the bottom of the cavity of the mouth. Fifty injections of methyl-violet solution 1 to 300, together amounting to 120 grammes, were made. After thirty-five injections the tumour shrivelled up, and the whole of the neoplasm lying outside of the bone had disappeared, reducing the original growth to one-third of its original size; subsequently it has become so much smaller that it may almost be said to be cured. Von Mosetig-Moorhof now uses a 1-500 solution, which is filtered through asbestos before it is used. About three to six grammes of this is injected at a time. No untoward effects have been observed, nor have any bad symptoms occurred from the entrance of the methyl-violet into the circulation. He summarises his results as follows: The injections cause complete cessation of neuralgic pains and slow and gradual diminution in the size of the tumours, by elimination in ulcerated growths, by softening, fatty degeneration, and absorption in masses covered with unaffected skin.

Masini, of Genoa, has also reported some experiments on the action of methyl-violet in diseases of the lungs, but his results are too vague and imperfect to warrant any conclusions as to the value of the dye in tubercular lung disease.

Professor Ehrlich and Dr. Lippemann have found that methylene blue, when perfectly free from chloride of zinc and other impurities, is a valuable and safe remedy for the relief of pain. They gave as much as fifteen grains in a day, in gelatine capsules, without any toxic symptoms being produced. The usual dose was from 1½ to 4 grains internally, and 1 grain hypodermically. It was employed successfully in rheumatism of the muscles and joints, and in various painful nervous diseases. It was very serviceable in two cases of migraine.

Lanc. Nov. 8, 1890; Rev. de Thérap. Med. Chir., Mar. 15, 1891; Boll. per le Mal. d. Oreach, Nasoe Gol. 6, 1890; Wein. Med. Woch. 6, 1891; All. Med. Zeit., Mar. 17, 1891; Gaz. d. Ospit., Mar. 11, 1891.

